# Defined, serum/feeder-free conditions for expansion and drug screening of primary B-acute lymphoblastic leukemia

**DOI:** 10.18632/oncotarget.22466

**Published:** 2017-11-15

**Authors:** Zhiwu Jiang, Di Wu, Wei Ye, Jianyu Weng, Peilong Lai, Pengcheng Shi, Xutao Guo, Guohua Huang, Qiuhua Deng, Yanlai Tang, Hongyu Zhao, Shuzhong Cui, Simiao Lin, Suna Wang, Baiheng Li, Qiting Wu, Yangqiu Li, Pentao Liu, Duanqing Pei, Xin Du, Yao Yao, Peng Li

**Affiliations:** ^1^ Key Laboratory of Regenerative Biology, Guangzhou Institutes of Biomedicine and Health, Chinese Academy of Sciences, Guangzhou 510530, China; ^2^ Guangdong Provincial Key Laboratory of Stem Cell and Regenerative Medicine, South China Institute for Stem Cell Biology and Regenerative Medicine, Guangzhou Institutes of Biomedicine and Health, Chinese Academy of Sciences, Guangzhou 510530, China; ^3^ Department of Abdominal Surgery, Affiliated Cancer Hospital and Institute of Guangzhou Medical University of Guangzhou Medical University, Guangzhou Medical University, Guangzhou, Guangdong 510095, China; ^4^ Department of Hematology, Guangdong Provincial People’s Hospital, Guangzhou 510500, China; ^5^ Department of Hematology, Nanfang Hospital, Guangzhou 510500, China; ^6^ Department of Respiratory Medicine, Nanfang Hospital, Southern Medical University, Guangzhou 510515, China; ^7^ Department of Hematology, The First Affiliated Hospital, Sun Yat-sen University, Guangzhou 510500, China; ^8^ The First Affiliated Hospital, University of Zhengzhou, Zhengzhou 450000, China; ^9^ Affiliated Caner Hospital and Institute of Guangzhou Medical University, Guangzhou 510095, China; ^10^ Department of Hematology, Medical College, Jinan University, Guangzhou 510632, China; ^11^ Wellcome Trust Sanger Institute, Hinxton, Cambridge CB10 1HH, England, UK

**Keywords:** B-ALL, microenvironment, growth factors, drug screening, kinase inhibitors

## Abstract

Functional screening for compounds represents a major hurdle in the development of rational therapeutics for B-acute lymphoblastic leukemia (B-ALL). In addition, using cell lines as valid models for evaluating responses to novel drug therapies raises serious concerns, as cell lines are prone to genotypic/phenotypic drift and loss of heterogeneity *in vitro*. Here, we reported that OP9 cells, not OP9-derived adipocytes (OP9TA), support the growth of primary B-ALL cells *in vitro*. To identify the factors from OP9 cells that support the growth of primary B-ALL cells, we performed RNA-Seq to analyze the gene expression profiles of OP9 and OP9TA cells. We thus developed a defined, serum/feeder-free condition (FI76V) that can support the expansion of a range of clinically distinct primary B-ALL cells that still maintain their leukemia-initiating ability. We demonstrated the suitability of high-throughput drug screening based on our B-ALL cultured conditions. Upon screening 378 kinase inhibitors, we identified a cluster of 17 kinase inhibitors that can efficiently kill B-ALL cells *in vitro*. Importantly, we demonstrated the synergistic cytotoxicity of dinaciclib/BTG226 to B-ALL cells. Taken together, we developed a defined condition for the *ex vivo* expansion of primary B-ALL cells that is suitable for high-throughput screening of novel compounds.

## INTRODUCTION

B-acute lymphoblastic leukemia (B-ALL) is a common form of cancer in children and adults. Approximately 80% of affected children can be cured with current chemotherapy regimens [1]. By contrast, treatment outcomes for adults with B-ALL are poor. Currently, the 5-year survival rate of adult B-ALL patients is less than 50% [2, 3]. Chemotherapy-resistant B-ALL cells are thought to be responsible for relapse and therapy failure [4]. To improve the outcomes and survival rates of both children and adults, there is an increasing need to introduce new approaches and more effective therapies.

Increasingly, studies suggest that the BM microenvironment contributes to the proliferation, survival and drug resistance acquisition of leukemic cells [5, 6]. MSCs are the major source of growth factors, which are essential for the survival and expansion of hematopoietic and leukemic cells [7]. It has been reported that hematopoietic growth factors, including SCF, IL-7, IL-4 and IL-3, support primary B-ALL cells *in vitro* [8, 9]. However, there is no consensus on the culturing methods for the *ex vivo* expansion of B-ALL cells. Thus, it is necessary to clarify the factors that can support the growth of primary B-ALL cells.

Protein kinases have considered as one of the most successful families of drug targets [10]. However, the long time-span from the discovery of kinases as oncogenes and key players in tumorigenesis to their clinical approval remains a major challenge for kinase chemical biology [11]. To date, a large number of selective kinase inhibitors have undergone clinical trials for cancer treatment [12]. Screening clinical kinase inhibitors would shorten the time and expenditure associated with drug development and would increase the feasibility of precision medicine.

Here, we demonstrated a defined, serum/feeder-free condition that supports the expansion of primary B-ALL cells *in vitro*. We confirmed that expanded B-ALL cells still maintain leukemia-initiating potential after being transplanted into immunodeficient mice. Based on primary B-ALL cell screening, we identified two kinds of kinase inhibitors (cyclin-dependent kinase (CDK) and PI3K inhibitors) that can efficiently inhibit the growth of B-ALL cells. Importantly, dinaciclib/BTG226 can synergistically induce cell death *in vitro* and enhance anti-tumor activity in patient-derived xenografts of B-ALL.

## RESULTS

### MSCs, not adipocytes, support the survival of human B-ALL cells

**Figure 1:**
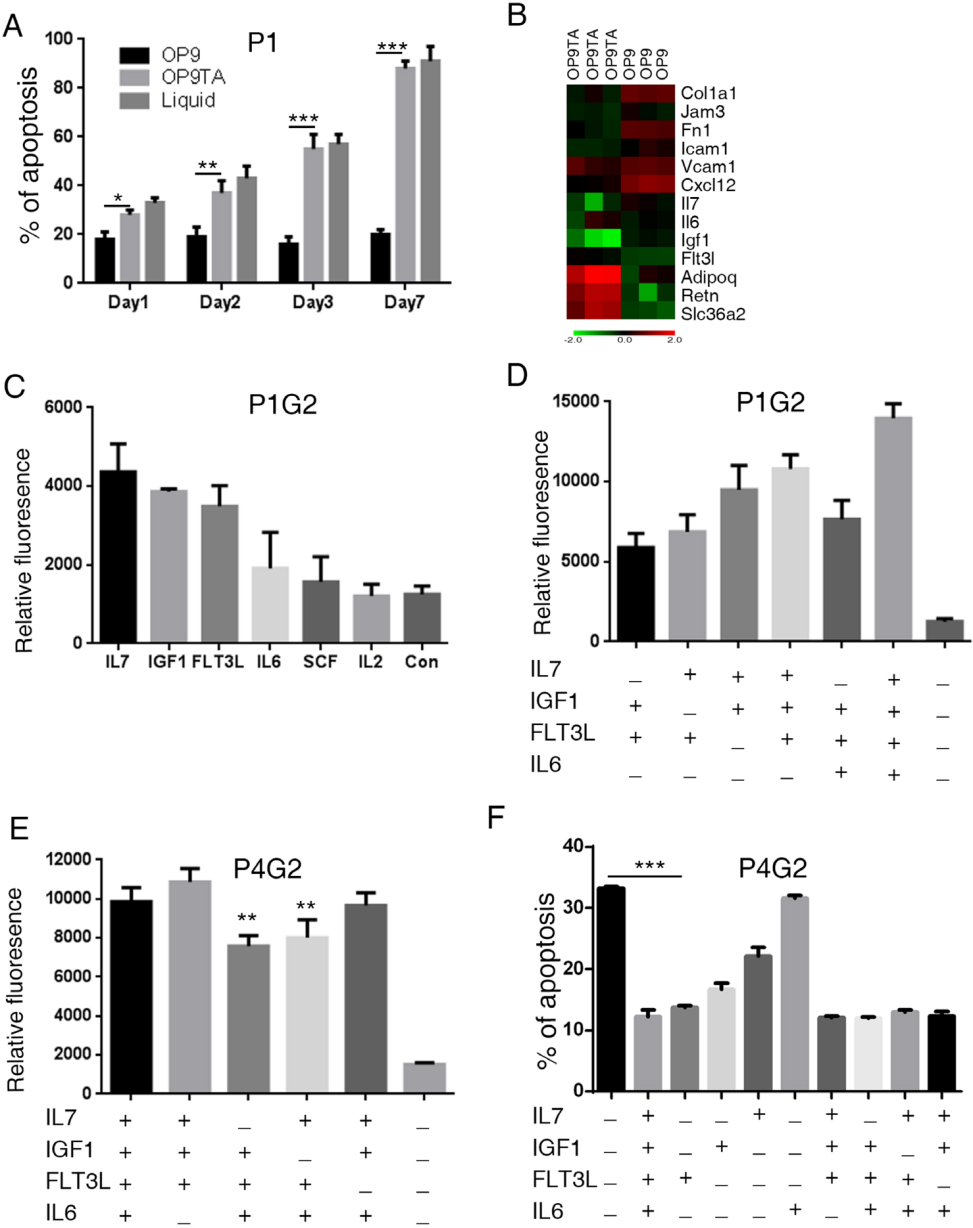
MSC-secreted growth factors maintain the activity of primary B-ALL cells *ex vivo* (**A**) Apoptosis of leukemic cells cultured *in vitro*. Human primary B-ALL (P1) cells were cultured in liquid (Culture medium supplemented with FBS) or co-cultured with OP9 and OP9TA cells for 1, 2, 3 and 7 days. Annexin V+ cells were counted as apoptotic cells. (**B**) Microarray analysis of adhesion and cytokine genes upregulated in OP9 cells compared with OP9TA cells. (**C**–**E**) B-ALL cells obtained from xenografts (P1G2 and P4G2) were seeded in 96-well plates at 1 × 10^5^ viable cells in 100 µl of IMDM with the indicated cytokines. Cell activities were measured 4–5 days later using a resazurin reduction assay. (**F**) The apoptosis of leukemic cells with the indicated cytokines added was measured by Annexin V staining after culturing the cells for 24 hours. The error bars indicates the S.D. of assays performed in triplicate. *p < 0.05, **p < 0.01, ***p < 0.001. P1 means leukemic cells directed from primary patient 1. P1G2 or P4G2 means leukemic cells from the second generation of xenografts of P1 and P4.

BM microenvironment mainly contains both MSCs and adipocytes. Previously, we showed that OP9, a bone marrow-derived MSC [13], supports the growth of primary B-ALL cells [14]. To investigate the effects of adipocytes on B-ALL cells, we induced OP9 cells to differentiate into adipocytes (Supplementary Figure 1A) and co-cultured primary B-ALL cells with these OP9-derived adipocytes (OP9TA). In the co-cultures, B-ALL cells adhered to OP9 cells and formed cobblestone-like features, but they did not bind to OP9TA cells (Supplementary Figure 1B). The apoptotic rate of B-ALL cells that were co-cultured with OP9 cells was approximately 20%. In contrast, the majority of B-ALL cells that were co-cultured with OP9TA cells or were in liquid culture underwent apoptosis (Figure 1A, Supplementary Figure 1C). These results show that MSCs, not adipocytes, maintain the survival of primary B-ALL cells.

### Growth factors produced by MSCs are necessary for B-ALL proliferation

We next examined the differential gene expression of OP9 and OP9TA cells (Supplementary Figure 2A, 2B) to investigate how OP9 cells support the survival of B-ALL cells. Analysis of the microarray data revealed that the expression levels of adherent proteins (Col1a1, Fn1, Jam3, Icam1, and Vcam1), cytokines and chemokines (Igf-1, Il-7, and Cxcl-12) were upregulated in OP9 cells (Figure 1B), whereas adipocyte-associated genes, including *Adipoq, Retn, and Slc36a2*, were upregulated in OP9TA cells (Figure 1B). These results were further confirmed by quantitative RT-PCR and FACS (Supplementary Figure 2C, 2D).

The growth factors secreted by MSCs have been shown to promote the growth of B-ALL cells. [15] Therefore, we compared the expression levels of growth factors in OP9 and OP9TA cells (Figure 1B) and found that OP9 cells highly express cytokines, mainly Igf-1 and Il-7 (Figure 1B). We then tested whether a combination of cytokines and membrane proteins could replace OP9 cells to support the expansion of primary human B-ALL cells in long-term culture. We screened the effects of various cytokines on primary human B-ALL cells and found that IL-7 and IGF-1 significantly stimulated the proliferation of B-ALL cells from patient #1 in liquid culture (Figure 1C). We also identified two other cytokines (FLT3L and IL-6) that had synergistic effects on promoting the expansion and survival of B-ALL cells (Figure 1D). Further optimization assays showed that a cocktail of IL-7, IL-6, IGF-1, and FLT3L (FI76) and a combination of IL-7, IL-6, and IGF-1 (I76) were better than other tested combinations at expanding primary B-ALL cells from three patients (Figure 1E, 1F and Supplementary Figure 3).

### Optimized serum-free conditions for *in vitro* culture of B-ALL cells

**Figure 2:**
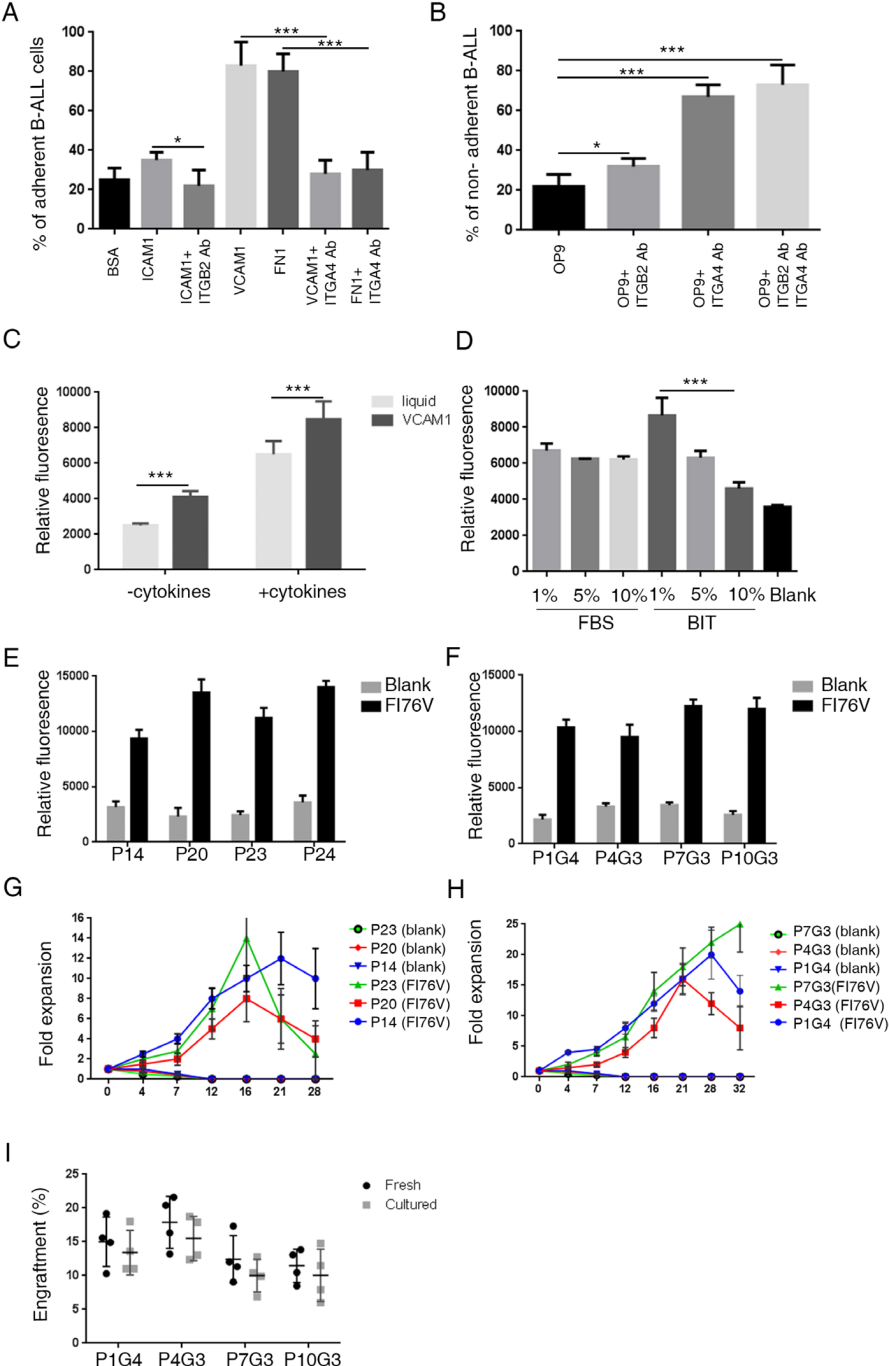
FI76V media supports robust growth of B-ALL cells *in vitro* and maintains leukemic-initiating cell capacity in mice (**A**, **B**) Representative adhesion of B-ALL cells from three patients to VCAM1 and fibronectin. B-ALL cells were seeded in 96-well plates at 1 × 10^5^ cells per well pre-coated with BSA, ICAM1, VCAM1 and FN1 or OP9 cells. Four hours after incubation, the cells suspended in the supernatant were removed and the plate was washed twice with culture medium. The adherent cells were detected using a resazurin reduction assay. The adhesion of leukemic cells to adhesion molecules and OP9 cells was inhibited by treatment with blocking antibodies against ITBG2 and ITGA4 (*n* = 3). (**C**) VCAM1 promotes the growth of B-ALL cells *in vitro*. A total of 1 × 10^5^ cells were cultured in 96-well plates with or without VCAM1 in IMDM with or without the indicated growth factors, and assays were performed 3 days later. The *ex vivo* growth of primary B-ALL cells was measured using a resazurin reduction assay. (**D**) The serum substitute supports the growth of B-ALL cells *in vitro*. (**E**–**H**) B-ALL cells obtained from primary patients and xenografts were seeded in VCAM1 pre-coated 24-well plates at 5 × 10^5^ cells per well in 1 ml of IMDM with or without cytokines. Every 3 days, half of the culture medium was replaced with fresh medium, and the cells were passaged every 7 days. Viable cell yields were counted with a hemocytometer. (**I**) Engraftment of fresh and cultured B-ALL cells in immunodeficient mice (*n* = 4 for each group). One million uncultured and cultured (for 3–4 weeks) B-ALL cells were transplanted into immunodeficient mice. FACS analysis shows the engraftment percentage of leukemic cells in the BM 4 weeks later. The error bars indicate the S.D. of assays performed in triplicate. *p < 0.05, ***p < 0.001.

To test whether VCAM1 or FN1 mainly mediate the adhesion of leukemic cells to OP9 cells, we plated human primary B-ALL cells into wells pretreated with VCAM1 or FN1 proteins. The results revealed that the adherence of B-ALL cells to culture plates was increased after the plates were coated with either VCAM1 or FN1 (Figure 2A, 2B). Consistently, we found that blockage of ITGA4 (ligand of VCAM1/FN1, Supplementary Figure 2E) by their antibodies significantly suppressed adhesion between OP9 and B-ALL cells (Figure 2A, 2B and Supplementary Figure 3E). These results suggest that human primary B-ALL cells bind to OP9 stromal cells mainly through interactions between VCAM1/FN1 and ITGA4. We further investigated whether adhesion molecules also promote B-ALL cell growth. We cultured primary B-ALL cells from xenografts in wells that contained FI76 medium and were pre-coated with VCAM1, and found that VCAM1 significantly promoted B-ALL cell growth compared with liquid conditions (Figure 2C).

Serum substitutes have been reported to maintain the viability of acute myeloid leukemia cells *in vitro* [10]. We investigated whether the serum substitute (BIT, 09500, Stem Cell Technologies) could replace fetal bovine serum (FBS). We compared the effects of FBS/BIT concentrations on B-ALL cell growth *in vitro*. Cells were maintained in IMDM supplemented with FI76V plus FBS/BIT. Interestingly, culture medium supplemented with 1% BIT significantly promoted B-ALL cell growth compared with culture medium supplemented with 10% BIT (Figure 2D). Based on these results, we cultured B-ALL cells in our optimized culture medium, referred to as FI76V, containing IMDM, 1% BIT, FLT3L (20 ng/ml), IGF-1 (10 ng/ml), IL-7 (10 ng/ml) and IL-6 (10 ng/ml) on VCAM1 pre-coated wells and evaluated the ability of the media to expand primary human B-ALL cells. B-ALL samples (Supplementary Table 1) obtained directly from patient biopsies and xenografts were seeded in FI76V media. In 19 cases tested, the B-ALL cells exhibited robust growth in FI76V media at first 2 weeks (Figure 2E, 2F). The cells were cultured in FI76V media, and the number of cells was counted at passage (every 4–7 days) for up to at least 4 weeks (Supplementary Table 2). The FI76V media maintained an expansion of 12 to 20 times (Figure 2G, 2H). Whole exome-sequencing analysis showed that B-ALL cells from *ex vivo* shared similar SNP profiles with the B-ALL cells that were freshly isolated from the same patients (Supplementary Figure 4A, 4B). Importantly, xenograft analysis confirms that these expanded B-ALL cells maintained the leukemia-propagating capacity (Figure 2I).

### Kinase inhibitors screening in primary B-ALL cells

**Figure 3:**
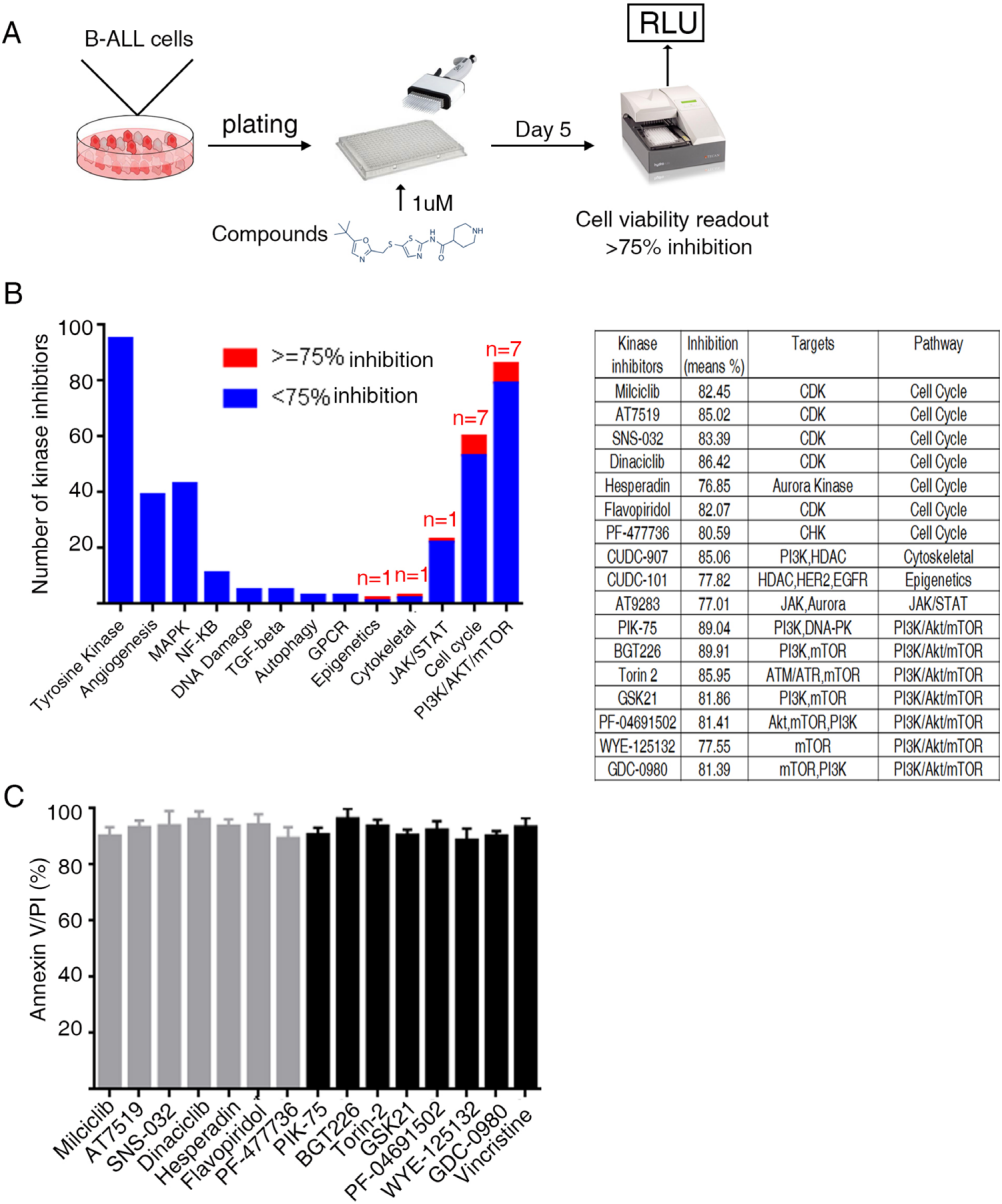
Primary B-ALL drug screens (**A**) Schematic outline of drug screening. Primary B-ALL cells were first expanded in FI76V conditions and then, plated in 384-well plates to which inhibitors had been added. Cell activities were measured 5 days later using a resazurin reduction assay. (**B**) The histogram shows B-ALL cell responses to various signaling pathway inhibitors. The red bar indicates that inhibition was above 75% (left). Information regarding the 17 kinase inhibitors that can efficiently inhibit the proliferation of B-ALL cells (right). (**C**) CDK and PI3K inhibitors (1 μM) induce apoptosis of primary B-ALL cells *in vitro* (*n* = 10).

We next confirmed whether the primary B-ALL cultured conditions were suitable for the screening of therapeutic compounds. A chemical screening was performed using a diverse library of 378 multi-targeted kinase inhibitors that had been tested in clinical therapy or cancer trial. Vincristine and daunorubicin, which are chemotherapy medications used to treat B-ALL, were used as positive controls. Inhibitors with above 75% inhibition of proliferation were selected (Figure 3A). Ten patients were used for compound screening. Our screening results show that vincristine and daunorubicin can efficiently suppress the growth of B-ALL cells in *ex vivo* (Supplementary Table 3). Seventeen kinase inhibitors with above 75% inhibition of cell proliferation were enriched. Further analysis revealed that these 17 inhibitors can be classified as CDK and PI3K pathway inhibitors (Figure 3B). The cytotoxicity of CDK and PI3K pathway inhibitors was further evaluated using Annexin V/PI staining followed by flow cytometry. B-ALL cells from 10 patients were incubated with 1 µM selected CDK and PI3K inhibitors for 24 hours. Our results revealed that these inhibitors efficiently induced apoptosis of B-ALL cells (Figure 3C). These results indicate the feasibility and credibility of our defined culture condition as a novel platform for high-through-put screening of drugs.

### Cytotoxicity of CDK/PI3K inhibitors to B-ALL

**Figure 4:**
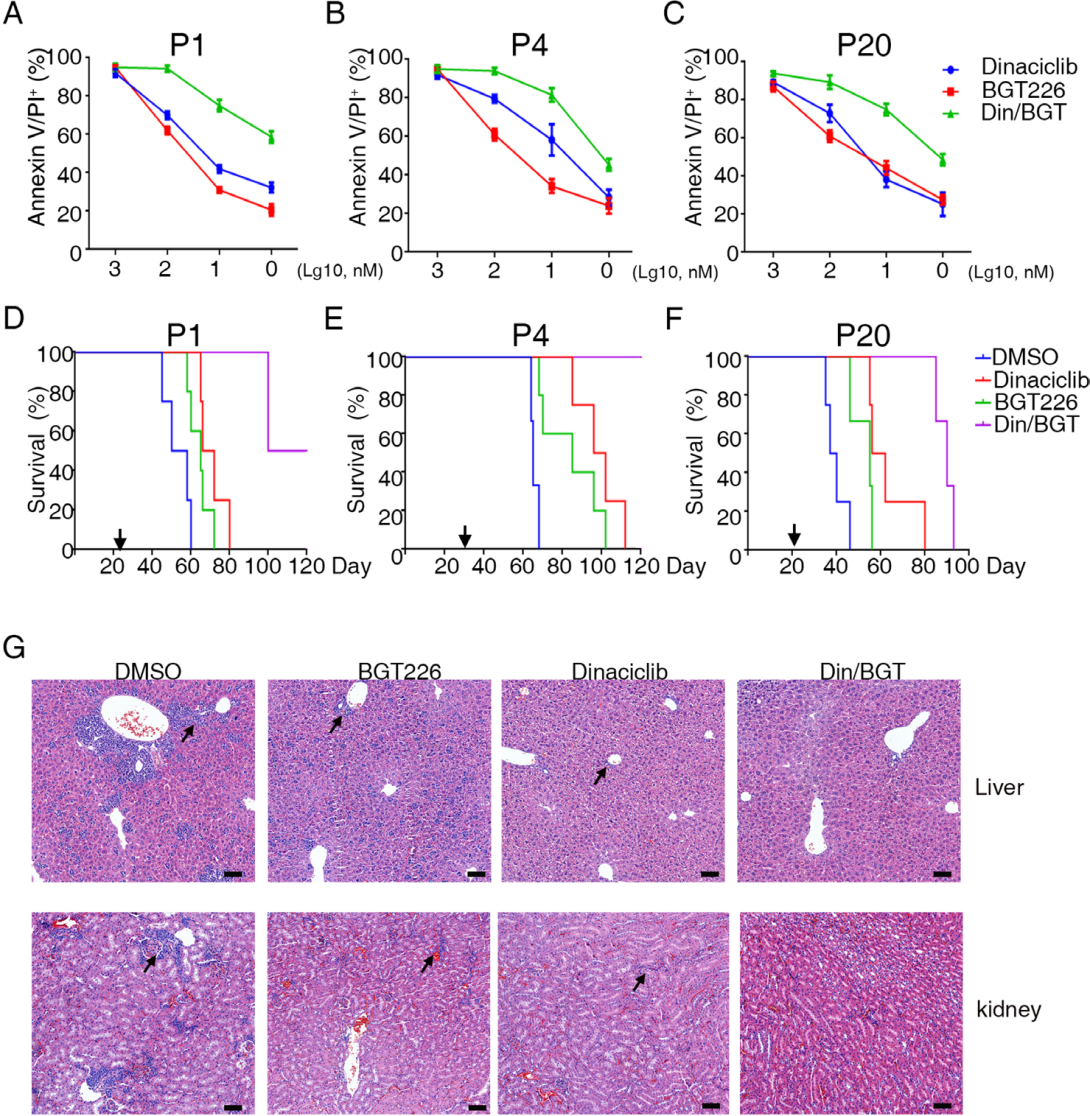
Dinaciclib and BGT226 induce the apoptosis of primary B-ALL cell *in vitro* and increase survival of xenografts (**A**–**C**) Primary B-ALL cells isolated from diagnosed patients were treated with dinaciclib, BGT226 and dinaciclib/BGT226 for 24 h, and the results revealed increases in Annexin V and PI-positive cells. (**D**–**F**) Kaplan-Meier plots of the survival curve are shown. NOD/SCID/IL2rg^–/–^ mice (*n* = 5) engrafted with the indicated human B-ALL samples were treated as shown when blasts in the peripheral blood reached >1% as indicated by the arrows below the plots. The error bars indicate the S.D. of assays performed in triplicate. (**G**) Representative H&E staining of livers and kidneys from mice treated with inhibitors. The migration of B-ALL cells into the liver and kidney was significantly suppressed by the dinaciclib/BGT226 combination. Scale bar = 50.

Previous studies had shown that a combination of CDK and PI3K inhibitors could efficiently inhibit the growth of breast and colorectal cancers in a xenograft tumor model [16, 17]. For further confirm the quality of above screening results, we further tested the cytotoxicity of CDK and PI3K inhibitors in primary B-ALL cells. Dinaciclib (a CDK inhibitor) and BTG226 (a PI3K inhibitor) which were more efficacy than others were selected (Figure 3B, 3C). The cytotoxicity of dinaciclib and BTG226 was further evaluated in primary B-ALL cells using Annexin V/PI staining followed by flow cytometry. Our results show that dinaciclib and BTG226 induced apoptosis in primary B-ALL cells in a concentration- and time-dependent manner (Figure 4A–4C and Supplementary Figure 3D) and combination of dinaciclib and BTG226 induced a substantial apoptosis than dinaciclib and BTG226 alone (Figure 4A–4C). This result was consistent with the recent reports that dinaciclib can efficiently kill AML cells [18] and that dinaciclib has a superior therapeutic index compared with both SNS-032 and flavopiridol in ovarian cancer [19]. Dinaciclib and BTG226 also induced apoptosis in NALM-6 and Reh cells in a concentration-dependent manner (Supplementary Figure 4C, 4D) and combination of dinaciclib and BTG226 induced more substantial apoptosis than dinaciclib or BTG226 alone (Supplementary Figure 4C, 4D). To examine the potential efficacy of dinaciclib and BTG226 inhibitors *in vivo*, we used patient-derived xenografts model of B-ALL. The NOD/SCID/IL2rg^-/-^ mice were engrafted with B-ALL cells and treated when 1% B-ALL cells were detected in the peripheral blood. Dinaciclib and BTG226 were used alone or combination at 10 mg/kg daily for 3 weeks. Both dinaciclib and BTG226 increased the overall survival of the mice from a median of 50 (range 40–64, *n* = 3 xenografts with 5 animals/group) days for control-treated mice to 76 (range 62.75–93.25, *n* = 3, *p* < 0.0001) days for dinaciclib-treated mice, 66 (range 57–78.5, *n* = 3, *p* = 0.0074) days for BGT226-treated mice and 96.5 (range 88.75–120.3, *n* = 3, *p* < 0.0001) days for dinaciclib/BGT226-treated mice (Figure 4D–4F). The histological results show that infiltrating leukemic cells in the livers and kidneys of the mice were inhibited in the dinaciclib/BTG226 combination group, and drug-induced liver and kidney injury were not observed (Figure 4G). Our results also show that treatment with a combination of dinaciclib and BTG226 significantly decreased the leukemia burden of NALM-6 cells in the mice (Supplementary Figure 4E, 4F) Together, our results indicate that CDK and PI3K inhibitors synergistically inhibited leukemia progress in both patient-derived and cell line xenografts of B-ALL.

## DISCUSSION

It has been reported that primary MSCs and growth factors support the growth and maintain the viability of B-ALL cells *in vitro* [15, 20]. These studies remind us that crosstalk between signaling pathways may play a role in maintaining the activity of B-ALL cells. However, to the best of our knowledge, a robust culture condition for expanding B-ALL cells is still not available. Our results show that MSCs actively anchor B-ALL cells to their cell surface through VCAM1/FN1-ICAM1 interactions. Additionally, we found that MSCs may support the proliferation of B-ALL cells mainly via paracrine signaling of IGF-1 and IL-7. The effects of adhesion and support of B-ALL cells disappeared after MSCs differentiated to adipocytes because adipocytes have decreased expression of VCAM1/FN1 and cytokines, such as IGF1 and IL7. It has been reported that interaction between VCAM1/FN1 and ITGA4 is essential for the survival of B-ALL cells [21]. In light of this, we optimized our culture conditions and found that FI76 and VCAM1 were essential for the survival and proliferation of B-ALL cells. Although not all primary B-ALL samples grow long term, primary B-ALL cells from 14 out of 19 patients have been successfully expanded approximately 10-fold for at least 4 weeks. This optimized culture condition will be more convenient and useful for drug screening and personalized drug sensitivity selection.

To date, tumor cell lines are widely used in laboratory research and are a valuable tool for investigating problems of clinical relevance. Ready access to cell lines makes them convenient to use for studies of molecular pathogenesis and drug screening. However, cell lines are prone to genotypic and phenotypic drift during continual culture, which may alter the original characteristics of the cell line [22, 23]. We first confirmed that primary B-ALL cells are also suitable for screening novel compounds in our derived B-ALL expansion conditions. We did drug screening for primary leukemia cells in conventional culture media (without cytokines). The results were un-reproductive which positive drugs (vincristine and daunorubicin) have less cytotoxicity to cells (data not shown). We propose that these primary leukemia cells undergo significant apoptosis which affect readout out of cytotoxicity of compounds. In this study, we finally identified 7 CDK inhibitors and 7 PI3K inhibitors that can significantly inhibit proliferation and induce apoptosis of B-ALL cells. CDKs and PI3K are key positive regulators of cell cycle progression. CDKs and PI3K inhibitors have been proposed as attractive drug targets and have been pursued for oncology indications for several years. Dinaciclib and BTG226 are currently undergoing clinical testing against a range of solid and hematologic malignancies [24, 25]. *In vitro* and *in vivo* studies presented in this study support the conclusion that dinaciclib and BTG226 have the potential to inhibit the growth of human B-ALL. Consistent with studies show that the combination of CDK/PI3K inhibitors enhance antitumor activity and overcome drug resistance in colorectal and breast cancers [16, 17], our results that the combination of dinaciclib/BTG226 can efficiently induce cell death and enhance *in vivo* anti-tumor activity in B-ALL cells. These results indicate that the CDK/PI3K inhibitors should be further investigated for use in the treatment of refractory/relapsed B-ALL.

In conclusion, our results demonstrate that MSCs, but not adipocytes, promote the growth of B-ALL cells through the synergistic effects of growth factors and adherent proteins. Therefore, we have determined a defined, serum/feeder-free condition that supports the growth of primary B-ALL cells *ex vivo*. We verified that dinaciclib/BTG226 can synergistically kill B-ALL cells *in vitro* and in xenografts. Taken together, we developed a defined condition for the *ex vivo* expansion of primary B-ALL cells that is suitable for high-throughput screening of novel compounds and precision medicine.

## MATERIALS AND METHODS

### Mice

Animal experiments were performed in the Laboratory Animal Center of the Guangzhou Institutes of Biomedicine and Health (GIBH), and all animal procedures were approved by the Animal Welfare Committee of GIBH. We generated NOD/SCID/IL2Rg^−/−^ mice by TALEN-mediated gene targeting in mice with a NOD background [14, 26]. All mice were bred and maintained in specific pathogen-free (SPF)-grade cages and provided with autoclaved food and water. The protocols were approved by the Institutional Animal Care and Use Committee (IACUC) at GIBH.

### B-ALL patient samples

All primary samples were obtained with informed consent for research purposes, and the procedures were approved by the Research Ethics Board of GIBH. BM and peripheral blood samples were obtained from B-ALL patients at the time of their initial diagnosis, which was based on cell morphology (FAB classification), cytochemistry, and routine immunophenotyping. All samples were collected at Nanfang Hospital. All related procedures were monitored and approved by the Institutional Review Boards of Southern Medical University. Mononuclear cells were separated by density gradient centrifugation (Lymphoprep, StemCell Technologies, Vancouver, BC, Canada). Samples were cryopreserved in liquid nitrogen in RPMI 1640 with 40% fetal bovine serum (FBS) and 10% dimethyl sulfoxide (DMSO) or were directly transplanted into immunodeficient mice. Mononuclear cells were obtained by centrifugation in density gradients and were washed twice in PBS. The clinical characteristics of the 25 patients are provided in previous studies [14].

### Cells and culture conditions

All cells were incubated at 37°C in a humidified atmosphere containing 95% air and 5% CO_2_. OP9 stromal cells (CRL-2749, ATCC, Manassas, VA, USA) were cultured in α-MEM (HyClone, Thermo Scientific, Waltham, MA, USA) supplemented with 20% fetal bovine serum (FBS) (Gibco, Thermo Scientific, Waltham, MA, USA), 2 mM L-glutamine, 100 U/ml penicillin, and 100 μg/ml streptomycin. NALM-6GL (acute lymphoblastic leukemia line, stably transfected with GFP and luciferase) and Reh (acute lymphoblastic leukemia line) cell lines were cultured in RPMI-1640 (Gibco, Life Technologies).

Primary B-ALL cells harvested from patients or xenografts were enriched via magnetic cell sorting using anti-human CD19 MicroBeads (Miltenyi Biotec, Germany) and cultured at a density of 1 × 10^6^/ml in FI76V condition including IMDM (HyClone, Thermo Scientific), 1% BIT (STEMCELL Technologies), 2 mM L-glutamine, 100 U/ml penicillin, and 100 μg/ml streptomycin with addition of 20 ng/ml FLT3L (Peprotech, 300–19), 10 ng/ml IGF-1(Peprotech, 100-11), 10 ng/ml IL-7 (Peprotech, 200-07), 10 ng/ml IL-6 (Peprotech, 200-07), as indicated results. Fresh medium were changed every three days.

### Kinase inhibitors screening

This assay was carried on white frame flat-bottom 384-well plates (Santa Cruz Biotechnology, Heidelberg, Germany). Compounds (10 mM in DMSO, Catalog No.L1200, Selleckchem) were diluted to a final concentration of 1 μM in the FI76V medium and automatically added to wells. The concentration of compounds for screening was referred to previous study [27]. Each compound was tested in triplicate. Primary B-ALL cell were seeded into the 384-well plate at 1 × 10^5^ cell per well and maintained in 200 μl chemically defined medium composed of IMDM (Invitrogen) supplemented with 1% BIT(STEMCELL Technologies), FLT3L, IGF-1, IL-7 and IL-6. Inhibitors response was analyzed 5 days later and cell growth was measured using a resazurin reduction assay (Cell Titer Blue, Promega).

### Xenotransplantation animal model

Xenograft-expanded B-ALL cells were injected intravenously via the retro-orbital route into NSI mice aged 8–10 weeks with preconditioning total body irradiation (TBI, 100 cGy). Mice were engrafted with B-ALL and treatment when 1% B-ALL was detected in the peripheral blood. DMSO (control), Dinaciclib (10 mg/kg, I.P.), BTG226 (10 mg/kg, I.P.) and Dinaciclib/BTG226 (5 mg/kg each, I.P.) were given five times weekly for 3 weeks. Groups of 6 mice received vehicle or inhibitors until sacrifice was required due to deterioration of health scores. For *in vivo* imaging, 5 × 10^5^ NALM6-GL cells were intravenously inoculated into NOD/SCID/IL2rg^−/−^ mice. At day 7, DMSO (control), Dinaciclib (10 mg/kg, I.P.), BTG226 (10 mg/kg, I.P.) and Dinaciclib/BTG226 (5 mg/kg each, I.P.) were given five times weekly for 2 weeks. The leukemia burden was evaluated using a cooled CCD camera system (IVIS 100 Series Imaging System, Xenogen, Alameda, CA, USA). The mice were injected intraperitoneally with d-luciferin firefly potassium salt at 75 mg/kg and then imaged 5 min later with an exposure time of 30 s. Quantification of the total and average emissions was performed using Living Image software (Xenogen).

### Histological analysis and immunofluorescence staining

Organ and tissue samples were fixed in 10% formalin, embedded in paraffin, sectioned at 4-μm thickness, and stained with hematoxylin and eosin. For immunofluorescence staining, cells were fixed with 10% formalin for 30 min, and were incubated with primary antibodies against ITGA4 (monoclonal mouse IgG1 clone#2B4, R&D Systems, Minneapolis, MN, USA) and ITGB2 (polyclonal goat IgG, R&D Systems, Minneapolis, MN, USA) overnight at 4°C. Then they were washed 3 times and incubated with Cy3-conjugated AffiniPure goat anti-mouse IgG (H+L) or Cy5-conjugated AffiniPure mouse anti-goat IgG (H+L) for an additional 60 min at room temperature. Finally, the cells were incubated with 4′,6-diamidino-2-phenylindole (DAPI) for 10 min at room temperature. Images were obtained on a Leica DMI6000B microscope (Leica Microsystems, Wetzlar, Germany).

### qRT-PCR

Total RNA was isolated from cells using TRIzol reagent (Invitrogen, Thermo Scientific, Waltham, MA, USA). cDNA was obtained with the SuperScript reverse transcriptase kit (TransGene, Beijing, China) and oligo-dT primers. All primers were synthesized by Invitrogen (Guangzhou, China). Gene expression was measured with the CFX96 Real-time PCR System (Bio-Rad, Hercules, CA, USA) using the SYBR Select Master Mix (Invitrogen, Thermo Scientific, Waltham, MA, USA) according to the manufacturer’s protocol.

### Flow cytometry

Flow cytometric analysis was performed with a FACSAria II cell sorter (BD Biosciences, San Jose, CA, USA). Approximately 1 × 10^6^ cells were stained with fluorescein-conjugated monoclonal antibodies against human leukocyte-surface antigens for 30 min at 4°C. Peripheral blood, spleen, and BM samples from the mice were processed according to standard procedures. FACS data were analyzed with FlowJo software (FLOWJO, LLC., Ashland, OR, USA).

### Microarray analysis

RNA was extracted with an RNeasy RNA Mini Kit (Qiagen, Stockach, Germany). The quality of the RNA was analyzed with an Agilent Bioanalyzer 2100 (Agilent, Santa Clara, CA, USA) before the next procedures. RNA was labeled and amplified according to the Affymetrix™ GeneChip Expression Analysis Technical Manual, and hybridized to the GeneChip Mouse Genome 430 2.0 Array. Chips were scanned using an Affymetrix GeneChip™ Scanner. The data were analyzed using dChip software (http://www.dchip.org). Gene expression data from OP9 and OP9TA cells were generated for this study.

### Whole-exome sequencing

Genomic DNA was extracted from indicated specimens using the DNeasy Blood & Tissue Kit (Qiagen, 69506) according to the manufacturer’s protocols. The concentration and quantity of total DNA were assessed by measuring absorbance with NanoDrop^®^ ND-1000 and runing 0.8% Argose gel. 6 ug of DNA was sheared and subject to whole-exome sequecing using the Agilent SureSelect All Exon capture probe set and sequenced by HiSeq2500. A median 7.9 Gb of unique sequence was generated for each sample. Sequence reads were aligned to the human reference genome buid 27, using Novoalign (novocraft.com).

### Induction of adipocyte differentiation

To induce adipocyte differentiation, cells were cultured as a monolayer. Upon reaching confluence, the cells were maintained for 2 more days and were then incubated in DMEM supplemented with 10% FBS, 1 µM dexamethasone, 0.5 mM isobutyl-methylxanthine, 5 µg/mL insulin and 1 µM Rosiglitazone (Sigma-Aldrich, St Louis, MO, USA) for 2 days. The medium was then replaced by DMEM plus 10% FBS and 1 µM dexamethasone for 7–14 days, with fresh medium every 2 days. Cells were fixed with 4% paraformaldehyde for 10 min, then stained with Nile red (Sigma-Aldrich).

### Cell adhesion assay

OP9 and OP9TA cells (1 × 10^5^) were seeded in 24-well plates and cultured for 1 day at 37°C to prepare confluent monolayers. After washing wells with culture medium, leukemic cells (1 × 10^6^) suspended in 0.2 ml culture medium were seeded into each well, and incubated for 2 hours at 37°C in a humidified atmosphere of 5% CO_2_. Non-adherent leukemic cells were recovered after each of 3 repetitions of vigorous agitation for 1 min. The recovered non-adherent leukemic cells were seeded in 96-well plates and detected by resazurin reduction assay. Ratios of the number of non-adherent leukemic cells relative to those initially added to each well were calculated.

The leukemic cells were also loaded in 96-well plates coated with or without human recombinant VCAM1 (hVCAM-1, 10 µg/ml) and fibronectin (hFN1, 25 µg/ml) (Sino Biological Inc.). After 2 hours’ incubation, cells suspended in the supernatant were removed and the plate was washed twice with culture medium. The adherent cells were detected by resazurin reduction assay.

For the assay with blocking antibodies, human primary B-ALL cells were either pretreated with blocking integrin alpha4 (ITGA4, 10 µg/ml) antibody (monoclonal mouse IgG1 clone #2B4, R&D Systems, Minneapolis, MN, USA) and integrin beta 2 (ITGB2, 20 µg/ml, polyclonal goat IgG, R&D Systems, Minneapolis, MN, USA) or control IgG1 (M5284, Sigma-Aldrich, St Louis, MO, USA) for 30 min at 37°C and washed once with PBS. Cells were then loaded in triplicate on 24-well plates with or without OP9 cells. After 2 hours’ incubation, cells suspended in the supernatant were removed and the plate was washed once with PBS. Non-adherent cells were seeded in 96-well plates and detected by resazurin reduction assay.

### Apoptosis assays

For apoptosis analysis, cells were stained with the Apoptosis Detection Kit (eBioscience, San Diego, California, USA). Briefly, cells were washed twice with PBS, suspended in 100 μl of binding buffer containing APC-conjugated Annexin V, and incubated in the dark for 15 min. Cells were then washed and suspended in 200 μl of binding buffer containing propidium iodide (PI). The percentage of apoptotic cells was quantified by FACS analysis.

### Statistical analysis

Data are presented as mean ± S.D. and were analyzed by IBM SPSS 20 with Student’s *t*-test. Multiple-group comparisons were performed using the One-way analysis of variance (ANOVA) followed by the Bonferroni posthoc test. Survival were estimated using the Kaplan-Meier analysis and compared using the log-rank test. Differences were considered statistically significant at *p* < 0.05.

## SUPPLEMENTARY MATERIALS FIGURES AND TABLES





## Abbreviations

B-ALL: B Acute lymphoblastic leukemia
OP9TA: OP9-derived adipocytes
CDK: cyclin-dependent kinase
FBS: fetal bovine serum
NSI: NOD/SCID/IL2Rg−/− mice
