# Supplementary material for: Defined, serum/feeder-free conditions for expansion and drug screening of primary B-acute lymphoblastic leukemia
Source: Oncotarget. 2017 Nov 15;8(63):106382–92. doi: 10.18632/oncotarget.22466 (PMC5739741; doi:10.18632/oncotarget.22466)
Supplement: Supplementary file 1 [file oncotarget-08-106382-s001.pdf]

Defined, serum/feeder-free conditions for expansion and drug screening of primary B-acute lymphoblastic leukemia

SUPPLEMENTARY MATERIALS

Supplementary Table 1: Clinical information of the 25 B-ALL patients

Patient ID	Sex	Age (year)	WBC (10^9/L)	LDH (U/L)	Spleno megaly	Risk	Status of d28	Xenografts
P1	Male	20	46.3	247.5	–	High	NR	Success
P2	Female	17	12.1	9022	+	High	NR	Success
P3	Male	58	135.51	1965.5	+	High	NR	Success
P4	Male	21	56			High	NR	Success
P5	Male	20	228.07	580.1	–	High	NR	No
P6	Male	47	100	434.4	–	High	NR	No
P7	Male	20	2.06			Standard	CR	Success
P8	Female	31	15.31	359.1	+	Standard	NR	No
P9	Male	40	47.4	533	–	High	NR	No
P10	Male	34	70.6	10000	+	High	NR	Success
P11	Female	38	80			High	NR	No
P12	Female	57	30	236	–	High	NR	No
P13	Female	19	10.89	1791	–	High	NR	Success
P14	Male	42	105.91	1135	+	High	NR	Success
P15	Female	25	30	207.8	–	High	NR	No
P16	Female	43	80	357.9	–	High	NR	No
P17	Male	19	99.62	285.9	+	High	NR	No
P18	Male	21	10.2	772.9	–	Standard	CR	No
P19	Male	35	66	184.4	+	High	NR	No
P20	Male	20	2.8	157	–	High	NR	Success
P21	Male	58	85.2	941	–	High	NR	No
P22	Male	32	20.2	1040.6	+	High	NR	No
P23	Male	21	4.9	213.7	–	Standard	CR	Success
P24	Female	19	237.8	1164.6	+	High	NR	Success
P25	Female	38	20.6			High	NR	Success

WBC: white blood cell,
Risk of adult B-ALL is based on age, WBC, immunophenotypes, and cytogenetic aberrations.
CR means complete-remission, NR means non-remission.
Xenograft success means that B-ALL can be reconstituted in NSI mice.

Supplementary Table 2: A summary of *in vitro* culture of primary B-ALL samples

Culture duration (weeks)	Basic medium Number (%), *n* = 19	FI76V Number (%), *n* = 19
0	19 (100)	19 (100)
1	19 (100)	19 (100)
2	7 (36.8)	17 (89.4)
3	2 (10.5)	16 (84.2)
4	0 (0)	14 (73.7)
5	0 (0)	11 (57.7)
6	0 (0)	9 (47.4)

B-ALL samples from 19 patients were cultured in FI76V conditions or basic media without cytokines. The viability of cultured B-ALL cells was subjected to statistics every week. 'Number (%)' indicates the number of B-ALL samples that were still viable at the indicated time point with expansion at least 2 folds.

Supplementary Table 3: The inhibtion effect of kinase inhibtiors in leukemic cells. See Supplementary_Table_3

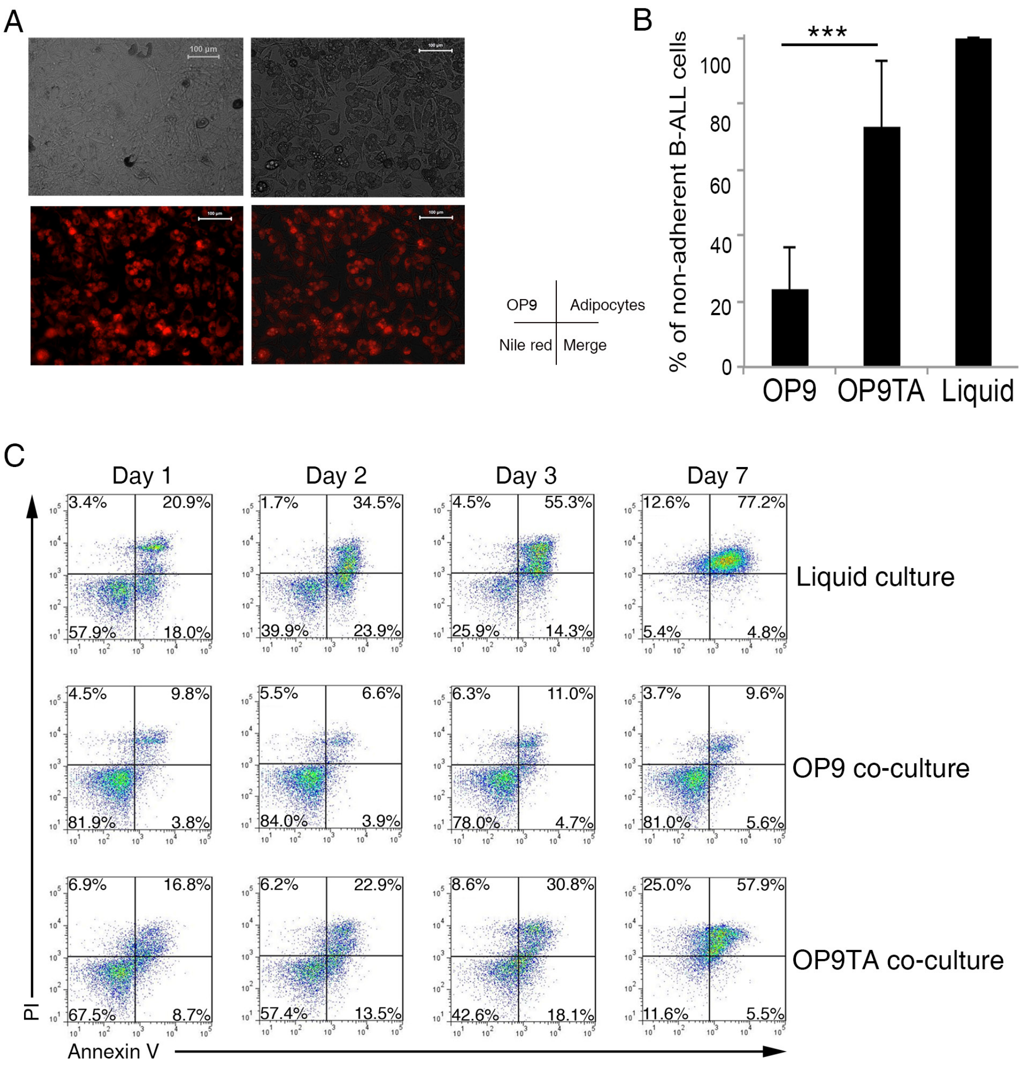

Supplementary Figure 1: Primary B-ALL cells gradually undergo apoptosis in liquid or OP9TA co-culture conditions. (**A**) Representative OP9 cell differentiation to adipocytes. Nile red stains the lipids (red color) in adipocytes. (**B**) The adhesion assay was performed on MSCs and adipocytes co-cultured for 24 hours. Percentages of non-adherent leukemic cells are indicated. (**C**) Representative FACS plots of primary B-ALL cells in various culture conditions. Cell apoptosis was detected by Annexin V and PI staining.

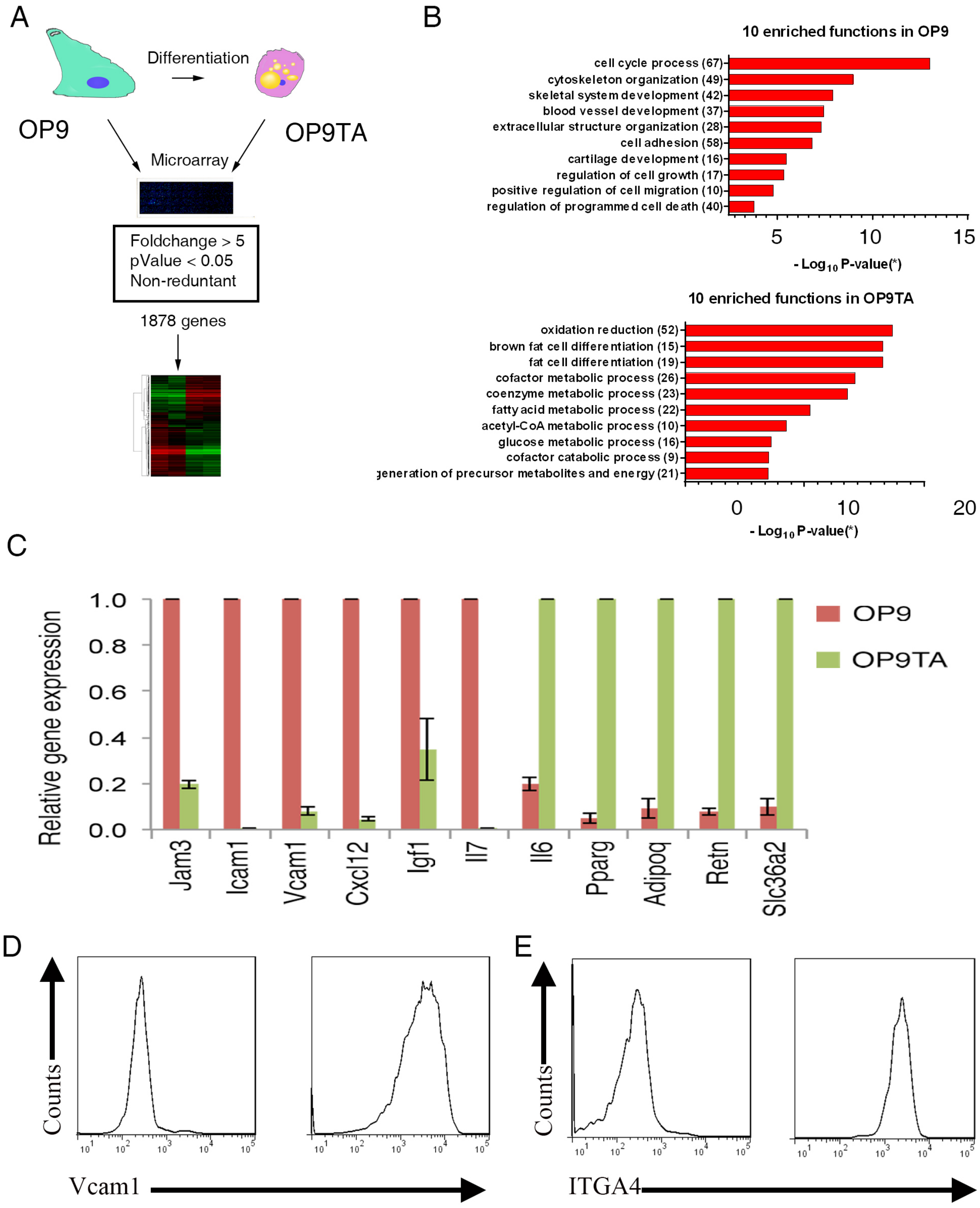

Supplementary Figure 2: Differential expression genes analysis in OP9 and OP9TA cells. (**A**) Working procedures of microarray analysis of transcriptome in OP9 and OP9TA cells. a greater than 5-fold change when $p < 0.05$ was considered to represent differential gene expression. (**B**) Gene set enrichment analysis (GSEA) in OP9 and OP9TA cells. (**C**) qRT-PCR validation of differentiated genes in OP9 cells compared with OP9TA cells. The error bar indicates the SEM of assays performed in triplicate. (**D**, **E**) FACS histograms show that vcam1 and ITGA4 expression in OP9 cells and primary B-ALL cells (P1), respectively.

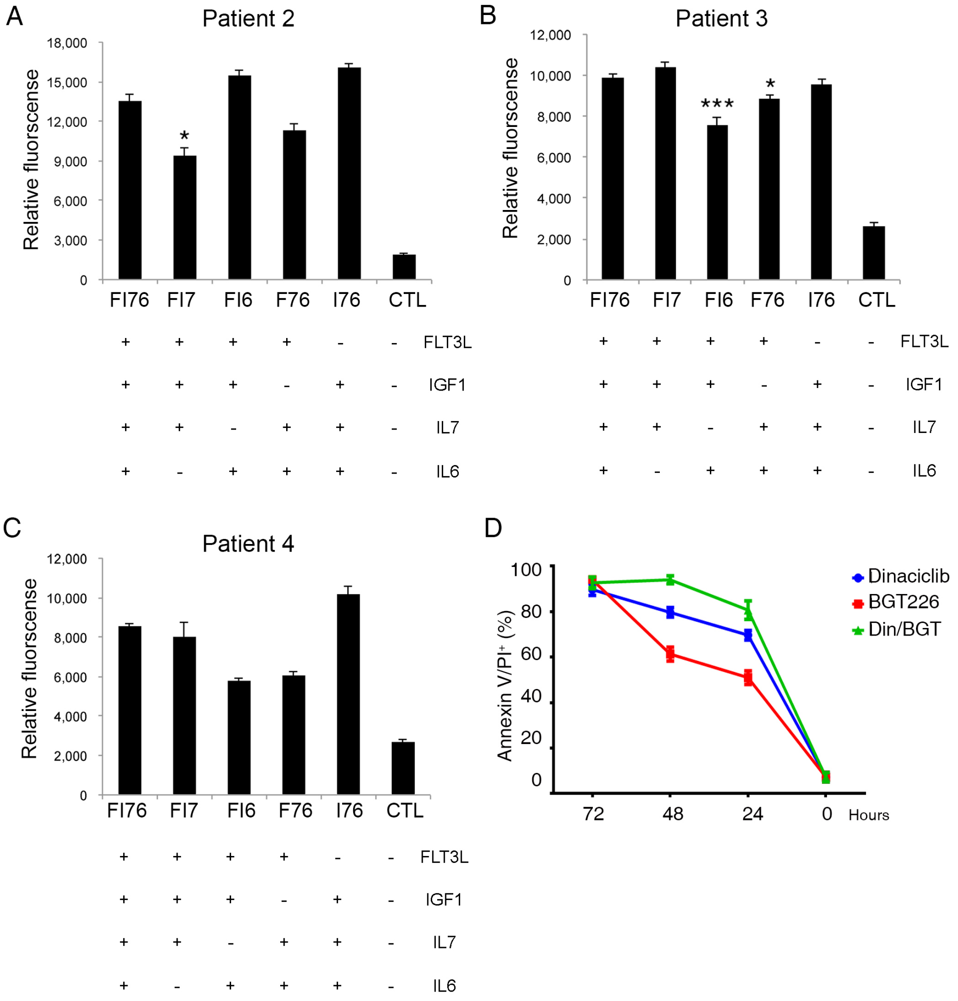

Supplementary Figure 3: Supplementary IGF-1, IL-7 and IL-6 are required for growth of primary B-ALL *in vitro*. *Ex vivo* growth of primary B-ALL cells was measured by resazurin reduction assay. 1×10^5 B-ALL cells from patient #1 (**A**), patient #2 (**B**), and patient #3 (**C**) were liquid cultured in 96-well plates with IMDM basic media lacking the indicated growth factors and assayed 5–7 days later. The error bar indicates the SEM of assays performed in triplicate. Statistical significance was calculated in comparison with the first column (FI76 group). $^{*}p < 0.05$, $^{**}p < 0.01$, $^{***}p < 0.001$. (**D**) Primary B-ALL cells isolated from diagnosed patients were treated with dinaciclib, BGT226 and dinaciclib/ BGT226 (100 nM each) for 24, 48 and 72 h, and showed increases in Annexin V and PI positive in time-dependence.

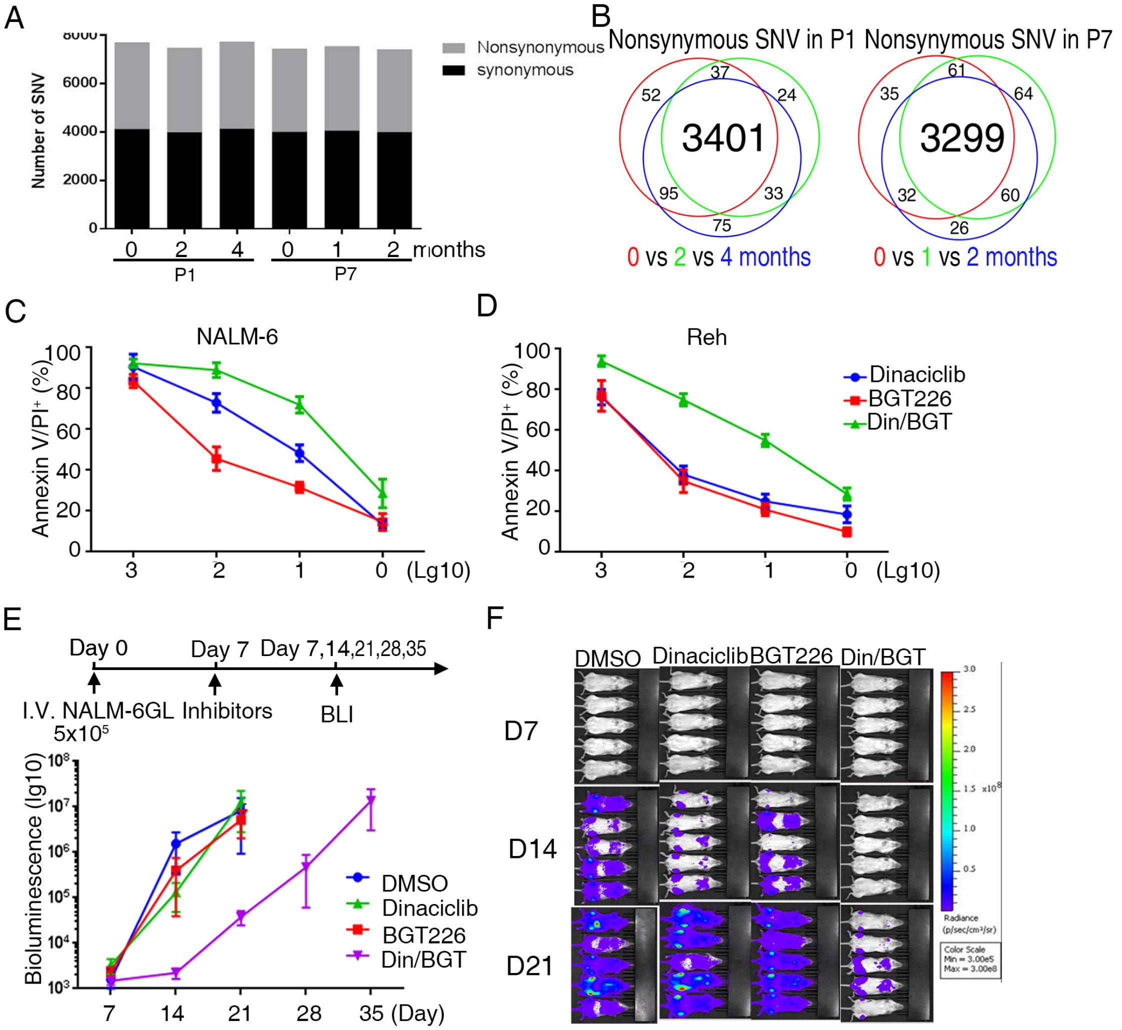

Supplementary Figure 4: Dinaciclib and BGT226 induce NALM-6 and Reh apoptosis and decrease leukemia burden in xenografts. (**A**) Whole exome sequencing of the leukemia in various *in vitro* culture durations revealed the presence of genome mutations. Similar rates of nonsynonymous and synonymous single nucleotide variants (SNVs) within the leukemia of patient1 and patient 7. (**B**) Venn diagram present B-ALL cells from various culture duration share similar nonsynonymous SNP profiles. (**C**, **D**) NALM-6 and Reh cell lines were treated with Dinaciclib, BGT226 and Dinaciclib/BGT226 for 24 h and showed increases in Annexin V and PI positive in dosage-dependence. (**E**) Timeline and events of the experiment with intravenous NALM-6GL xenograft models. On day 0, 5×10^5 NALM-6GL cells were injected through the tail vein into the NSI mice ($n = 5$), followed by treatment with Dinaciclib and BGT226. Tumor bioluminescence as mean photon count with standard deviations of mice groups. (**F**) Serial tumor bioluminescence imaging of mice at indicated time points.
